# Preventing ventilator-associated pneumonia via the gut-lung axis: the Shenling Baizhu San hypothesis

**DOI:** 10.3389/fmed.2026.1829153

**Published:** 2026-06-26

**Authors:** Xinyu Zhang, Jiawen Li, Yujia Lai, Guanxi Ren, Saiyin Chaoketu, Guofeng Cai

**Affiliations:** 1 Second Clinical Medical College, Heilongjiang University of Chinese Medicine, Harbin, China; 2 Department of Wu-Liao and Rehabilitation, International Mongolian Hospital of Inner Mongolia, Hohhot, China; 3 Hanan Branch of the Second Affiliated Hospital of Heilongjiang University of Chinese Medicine, Harbin, China

**Keywords:** gut microbiota, gut-lung axis, intestinal barrier, Shenling Baizhu San, short-chain fatty acids, ventilator-associated pneumonia

## Abstract

Ventilator-associated pneumonia (VAP) is a common hospital-acquired infection in the intensive care unit (ICU). It is associated with high morbidity and mortality, which are often compounded by patient frailty and disease severity. Despite adherence to antibiotic treatment guidelines, mortality rates remain persistently high. Critical illness disrupts intestinal function and alters epithelial cell dynamics, specifically increasing apoptosis and decreasing proliferation, thereby compromising the homeostasis of the epithelial monolayer. This increased intestinal permeability facilitates the translocation of bacteria and microbial products. Furthermore, impaired intestinal immunity exacerbates systemic inflammation and organ dysfunction. Previous studies indicate that the early onset of severe acquired immunosuppression in ICU patients significantly increases the risk of secondary infections. The gut-lung axis, which involves bidirectional crosstalk between the gastrointestinal and respiratory systems, is closely linked to immune regulation and the progression of lung diseases. We hypothesize that Shenling Baizhu San (SLBZS), a polysaccharide-rich multi-herb formula traditionally used for gastrointestinal disorders, could reduce susceptibility to VAP by reshaping the gut microbiota, enhancing the intestinal mucosal barrier, and modulating the transition from systemic inflammation to critical illness-induced immunoparalysis (CIIP) through microbiota-derived metabolites such as short-chain fatty acids (SCFAs). Validating this hypothesis would provide a novel, integrative therapeutic strategy for managing high-risk VAP patients.

## 1 Introduction

Ventilator-associated pneumonia (VAP) is a parenchymal lung infection that occurs in patients receiving invasive mechanical ventilation for at least 48 h. It remains one of the most common intensive care unit (ICU)-acquired infections and is associated with substantial mortality (commonly reported in the 20–50% range) (1). Despite evidence-based management guidelines, VAP remains difficult to prevent and treat, and reported incidence varies widely (5–40%), in part because diagnostic and surveillance definitions differ across settings (1–3). This heterogeneity complicates comparisons across ICUs and limits the generalizability of clinical trials.

Critical illness precipitates severe gastrointestinal disturbances. Factors such as intestinal hypoperfusion, shock, systemic inflammation, immunodeficiency, dietary changes, and reduced motility contribute to gut microbiota dysbiosis (4). Up to 90% of commensal gut microbiota can be depleted within 6 h of ICU admission (5), shifting the microbiome toward lower diversity and a preponderance of opportunistic pathogens (5). Such profound disruptions to the microbiota, epithelial integrity, and intestinal permeability may trigger systemic inflammation and organ dysfunction, while increased permeability facilitates the translocation of bacteria and endotoxins (6).

The gut-lung axis represents a bidirectional communication pathway linking the gastrointestinal and respiratory systems. Dysbiosis within these systems is closely linked to the pathogenesis and progression of pulmonary diseases (7, 8). Consequently, modulating the gut microbiota, its metabolites (e.g., short-chain fatty acids, SCFAs), and barrier function offers a therapeutic strategy for lung disease (8). For example, survivor-non-survivor differences in gut microbiota composition have been observed in severe community-acquired pneumonia, supporting an association between dysbiosis and adverse outcomes (9). In critical illness, factors such as antibiotic exposure, enteral feeding intolerance, and systemic inflammation jointly drive dysbiosis and barrier disruption. This increases microbial translocation and reshapes systemic immune trajectories, exacerbating secondary infection risks. Within this framework, ICU-feasible enteral adjuncts provide a straightforward translational route to target gut-lung axis biology.

Taken together, these observations provide a mechanistic rationale for testing enteral, gut-targeted adjuncts to reduce susceptibility to secondary pulmonary infections in the ICU. Shenling Baizhu San (SLBZS) is a classic multi-herb oral botanical formulation with growing experimental evidence for gut-targeted effects, including modulation of epithelial barrier function and the gut microbiota, with downstream effects on endotoxin burden and inflammatory responses (10–12). Therefore, we hypothesize that enteral SLBZS, as an adjunct to standard ICU care, could improve gut ecosystem stability and barrier integrity, support immune competence, and ultimately reduce susceptibility to VAP. This hypothesis yields falsifiable predictions that can be evaluated using gut-lung axis-relevant biomarkers and immune-trajectory measures linked to secondary infection risk; if supported, it would motivate a feasible enteral, gut-directed adjunct strategy for VAP prevention.

## 2 The hypothesis

### 2.1 Rationale: targeting the gut-lung axis to reduce VAP risk

The gut-lung axis is a bidirectional communication network mediated by microbial products, metabolites, and mucosal immune signaling (8, 13). In critically ill patients, disruption of intestinal barrier integrity and rapid depletion of commensal taxa can increase microbial-product translocation and alter systemic immune responses, which may weaken host defense and increase vulnerability to secondary infections, including VAP (6, 14, 15).

Current VAP prevention and management strategies largely emphasize airway- and aspiration-related pathways and infection-prevention bundles; however, ICU-acquired dysbiosis and barrier failure may represent an under-addressed upstream contributor to infection susceptibility. This perspective motivates a complementary enteral strategy that targets gut-lung axis biology at the bedside.

This hypothesis evolved from converging observations that (i) critical illness and common ICU exposures are associated with rapid dysbiosis and barrier dysfunction linked to higher secondary infection risk (6, 14, 15), and (ii) gut-directed interventions can modulate microbiota-derived metabolites such as SCFAs, which have been implicated in shaping systemic immune trajectories and pulmonary immune tone (13, 16, 17). We therefore propose that preserving intestinal barrier function and microbiome resilience may help mitigate critical illness-induced immunoparalysis (CIIP) and, in turn, reduce susceptibility to VAP.

### 2.2 Mechanistic hypothesis

**Figure 1:**
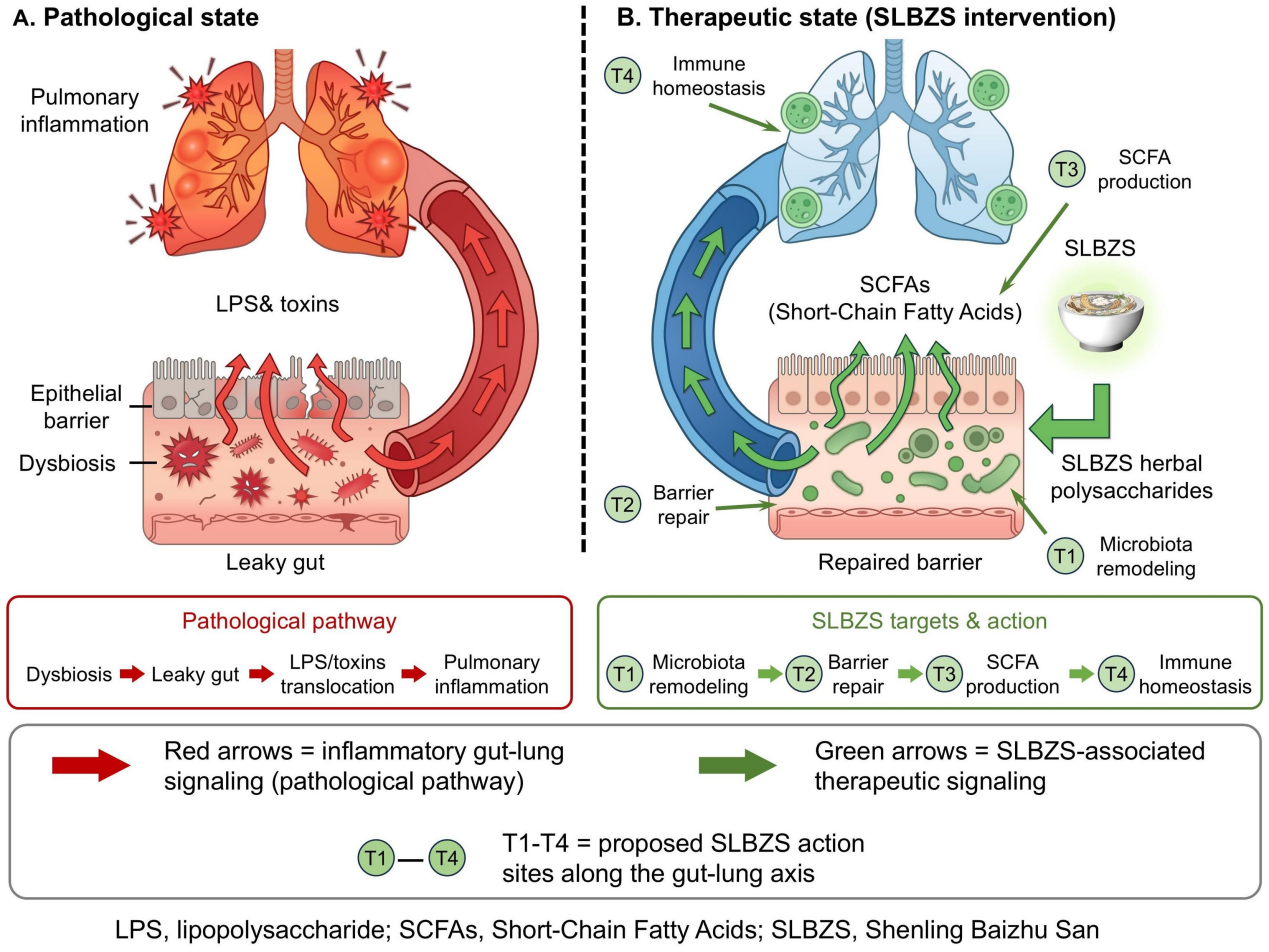
Hypothetical mechanism of SLBZS in reducing VAP susceptibility via the gut-lung axis. **(A)** Pathological state. Critical illness and antibiotic exposure induce gut microbiota dysbiosis and epithelial barrier disruption (leaky gut). These changes promote LPS translocation and systemic inflammation, which may impair pulmonary host defense and increase VAP susceptibility. **(B)** Therapeutic state. SLBZS is hypothesized to act through four gut-lung axis targets: T1, remodeling of the gut microbiota; T2, repair of the epithelial barrier; T3, increased production of SCFAs; and T4, systemic immune modulation supporting immune homeostasis. These coordinated effects may attenuate inflammatory gut-lung signaling and reduce susceptibility to VAP.

We hypothesize that, in selected ICU patients expected to require invasive mechanical ventilation for more than 48–72 h, enteral SLBZS added to standard care may reduce VAP susceptibility (Figure 1). VAP is typically triggered by microaspiration and airway-related factors, but susceptibility is shaped by systemic host defenses and immune trajectory. We conceptualize a complementary, gut-centered cascade that may contribute to VAP vulnerability (3, 14, 15, 18, 19): (i) ICU stressors and exposures (e.g., critical illness physiology and antibiotics) disrupt the gut ecosystem; (ii) epithelial injury and dysbiosis develop, with impaired barrier integrity and loss of colonization resistance; (iii) translocation of microbial products (e.g., lipopolysaccharide [LPS]) into the circulation increases; (iv) systemic immune responses become dysregulated and may progress toward CIIP; and (v) pulmonary host defense is compromised, increasing the likelihood that microaspiration events progress to infection and VAP. We propose that SLBZS primarily intervenes at steps (ii)–(iv) through three biological actions:

1. Restoration of intestinal barrier integrity. Critical illness triggers epithelial apoptosis and tight junction degradation, creating the leaky gut that allows the systemic spillover of pro-inflammatory ligands (e.g., LPS) (14). We propose that SLBZS acts as a structural barrier-repair agent, actively upregulating tight junction proteins to seal paracellular pathways. This physical restoration improves barrier function and reduces endotoxin burden, thereby attenuating the propagation of downstream systemic inflammation and CIIP (3, 14).
2. Metabolic recovery via support of SCFA-producing commensals. Antibiotic exposure and dietary shifts can deplete commensal diversity, including SCFA-producing bacteria (14). We hypothesize that the polysaccharide-rich, fermentable components of SLBZS act as prebiotic-like substrates. By resisting upper gastrointestinal digestion, they support the recovery of SCFA-producing taxa and robustly increase systemic SCFA levels. These metabolites can enter the circulation and shape distal immune tone, including in the lung (16, 17).
3. Priming of pulmonary immune competence. Gut-derived SCFAs serve as crucial signaling molecules for the distal immunomodulation of the lung. We propose that these circulating metabolites modulate alveolar macrophage function, limiting excessive inflammation while supporting antimicrobial effector functions (13, 16). By favoring a balanced immune recovery rather than persistent hyper-inflammation or subsequent CIIP, SLBZS may improve pulmonary clearance of microaspiration-related pathogens and ultimately reduce VAP risk (3, 18).
4. Stage-dependent immune modulation. The term “immune regulation” in this hypothesis does not imply non-specific immune stimulation. Critical illness often evolves from an early hyperinflammatory phase toward CIIP, although these states may overlap within the same patient (18). We propose that SLBZS may exert stage-dependent immunomodulatory effects: during early systemic inflammatory response syndrome, improved barrier integrity and reduced microbial-product translocation may attenuate excessive inflammatory amplification; during the later CIIP-prone phase, restoration of microbiota-derived metabolites such as SCFAs may support immune homeostasis and partial recovery of antimicrobial competence. However, patients with profound CIIP may have a limited response if their gut microbiota lacks sufficient fermentative capacity. To avoid inappropriate immune activation, future trials should stratify or monitor patients using immune markers such as monocytic HLA-DR (19, 20), lymphocyte counts, cytokine profiles, and infection status, and should predefine stopping rules for unexpected inflammatory deterioration.

## 3 Evaluation of the hypothesis

### 3.1 The gut-lung axis as a pathobiological driver in VAP

The functional connectivity between the gastrointestinal and respiratory systems provides the theoretical foundation for our hypothesis. In critically ill patients with severe pneumonia or prolonged mechanical ventilation, the gut microbiota undergoes profound rapid dysbiosis, characterized by the depletion of beneficial commensals and the expansion of potentially pathogenic taxa (21). Mechanistic syntheses indicate that dysbiosis, coupled with increased intestinal permeability, can promote systemic immune dysregulation through altered metabolite signaling and the dissemination of pro-inflammatory ligands (22–24). Collectively, these gastrointestinal alterations may compromise pulmonary host defenses, rendering the host more permissive to secondary respiratory infections such as VAP (15). Recent clinical studies have strengthened the biological plausibility of gut-lung interactions in severe respiratory disease. In a 2026 prospective matched case–control study of 100 infants and young children with severe pneumonia, sepsis was associated with reduced gut microbial diversity, suggesting that gut microbial signatures related to endotoxin production and SCFA metabolism may help identify high-risk pneumonia (25). Another 2026 multi-omics study in hospitalized SARS-CoV-2 patients demonstrated that integrated respiratory and intestinal microbiome profiling, together with PBMC transcriptomics and clinical indicators, could stratify the risk of pulmonary fibrosis progression (26). However, none of these studies directly tested SLBZS or microbiota-based therapy for VAP prevention; therefore, they should be interpreted as supportive evidence for feasibility and biological plausibility rather than proof of efficacy.

### 3.2 Evidence map and ICU-specific translational boundary

Because no published clinical trials or preclinical studies in major international databases (e.g., PubMed, Web of Science) have directly evaluated SLBZS for VAP prevention in mechanically ventilated ICU patients, the supporting evidence can be organized into three levels. First, ICU studies have demonstrated that critical illness, antibiotic exposure, impaired motility, shock, and enteral feeding intolerance are associated with gut dysbiosis, intestinal barrier disruption, microbial translocation, and secondary infection risk. Critically ill patients receiving invasive mechanical ventilation frequently develop leaky gut phenotypes and enteral feeding intolerance (27). In analogous models of intestinal stress, such as ulcerative colitis (UC) with systemic inflammatory features, SLBZS treatment has been shown to modulate the structure and function of the intestinal flora and restore the abundance of beneficial bacteria (28, 29). Second, in preclinical models, whole-formula SLBZS has been reported to upregulate tight junction proteins such as zonula occludens-1 (ZO-1) and occludin, in association with a reduced endotoxin burden (10). Extrapolating this restorative capacity to the ICU setting suggests that SLBZS could mitigate critical illness-associated hyperpermeability and dysbiosis, thereby potentially reducing upstream triggers that propagate systemic inflammation. Third, the proposed link between SLBZS-induced gut restoration and reduced VAP susceptibility remains inferential and requires direct validation in ICU-specific models and clinical studies.

Several ICU-specific features may limit the extrapolation of existing preclinical findings. Mechanically ventilated ICU patients often experience hemodynamic instability, intestinal hypoperfusion, vasopressor exposure, sedative-and opioid-induced gastrointestinal dysmotility, broad-spectrum antibiotic pressure, proton pump inhibitor use, altered enteral nutrition, renal or hepatic dysfunction, and multi-organ failure. These factors may reduce the viability of commensal fermenters, impair SCFA production, alter herbal pharmacokinetics, and increase the risk of feeding intolerance. Therefore, the effects of SLBZS should be tested under ICU-relevant conditions before clinical implementation in mechanically ventilated patients. Future work should first determine whether the ICU gut ecosystem retains sufficient metabolic capacity to ferment SLBZS-derived polysaccharides and whether enteral SLBZS can be safely delivered during common ICU exposures. These ICU specific factors do not negate the biological rationale of the SLBZS-gut-lung axis, but they define the translational boundary that must be addressed before clinical implementation.

### 3.3 Component-level mechanisms: unpacking the SLBZS gut-lung action

Rather than acting through a strictly sequential chronological pathway, critical illness-associated gastrointestinal dysfunctions—barrier failure, microbial dysbiosis, and CIIP—occur concurrently and may continuously amplify one another through positive feedback loops. Accordingly, to validate our mechanistic hypothesis, we evaluate how the key ingredients of SLBZS (*Atractylodes macrocephala*, *Poria cocos*, and *Panax ginseng*) act to target this integrated pathological network:

1. *Atractylodes macrocephala* (Barrier repair). A foundational element in mitigating gut-origin systemic inflammation is counteracting epithelial hyperpermeability. *Atractylodes macrocephala* polysaccharides function as structural reinforcements. Evidence across multiple intestinal injury models indicates that these polysaccharides can preserve epithelial integrity by upregulating tight junction proteins, specifically ZO-1 and occludin (30). By sealing paracellular pathways, *Atractylodes* may limit the systemic dissemination of luminal microbial products (e.g., LPS) that could otherwise contribute to persistent inflammatory signaling and downstream CIIP (16).
2. *Poria cocos* (Ecological and metabolic support). Operating in tandem with barrier structural repair is the need to reverse ecological collapse. *Poria cocos* acts as a prebiotic-like substrate within the formulation. *In vitro* and *in vivo* studies indicate that *Poria* polysaccharides resist upper gastrointestinal digestion, reaching the colon to undergo targeted fermentation (31). This process shifts the microbial community toward beneficial taxa (e.g., *Lactobacillus* and *Bifidobacterium*) while suppressing pathobionts (e.g., *Escherichia-Shigella*) (31). Crucially, this ecological rescue can increase the output of SCFAs, providing a plausible metabolite-mediated route for gut-lung crosstalk.
3. *Panax ginseng* (Synergistic fermentation and immunomodulation). The systemic effector arm of this hypothesis links gut-level modulation to pulmonary protection via a dual-action gut-lung-metabolite axis. First, ginseng polysaccharides may act synergistically with *Poria* as fermentable substrates, potentially amplifying circulating SCFA profiles (32). Second, and relevant to distal defense, ginseng bioactives (such as ginsenosides) may work in concert with gut-derived metabolites. Upon reaching the lung via the circulation, this integrated metabolite profile has been reported in experimental contexts to modulate pulmonary inflammatory signaling pathways, including nuclear factor (NF)-κB-related mechanisms (16, 33, 34). In addition, SCFAs have been linked in experimental settings to altered alveolar macrophage phenotype that can balance inflammatory restraint with maintenance of antimicrobial effector functions (e.g., pathogen clearance) (35–37). Thus, ginseng may translate enteral therapy into distal respiratory protection by helping stabilize systemic immune trajectories away from persistent dysregulation or CIIP and toward balanced recovery, thereby reducing the likelihood that microaspiration-related exposures progress to infection and VAP.

Beyond the primary triad, the remaining SLBZS components reinforce this axis through complementary mechanisms. *Dioscorea opposita* and *Coix lacryma-jobi* expand the prebiotic substrate pool for robust SCFA production (38, 39). Concurrently, *Amomum villosum* restores gastrointestinal motility, mitigating ICU feeding intolerance and stasis-induced bacterial translocation (6, 40). At the terminal respiratory site, *Platycodon grandiflorus* enhances mucociliary clearance to physically expel microaspirated pathogens (41). Collectively, these botanicals seamlessly bridge enteral metabolic recovery with direct mechanical airway protection.

### 3.4 Critical evaluation of counter-arguments and limitations

Establishing the clinical validity of this hypothesis requires acknowledging its limitations and apparent contradictions within the ICU environment:

1. The antibiotic paradox. The most formidable challenge to our hypothesis is the ubiquitous use of broad-spectrum empirical antibiotics in ventilated patients. Because SLBZS relies on commensal bacteria to ferment its polysaccharides into active SCFAs, a decimated ICU microbiome could theoretically render the formulation inert (14, 17, 42). However, emerging evidence suggests that specific complex polysaccharides can provide a competitive advantage to resilient SCFA-producing taxa, accelerating their recovery even during or immediately following antibiotic exposure (43–45). This implies that SLBZS might be most effective when administered precisely as an early prophylactic adjunct to preserve commensal resilience, rather than as a late-stage rescue therapy (46).
2. The bioavailability limitation. Pharmacological critiques often highlight the poor systemic absorption of high-molecular-weight botanical polysaccharides. However, viewed through the lens of the gut-lung axis, this low bioavailability is mechanistically essential. These macromolecules must remain unabsorbed in the small intestine to serve as fermentation substrates in the colon. The active therapeutic agents reaching the lungs are not the intact polysaccharides, but the immunoregulatory SCFAs they generate (17).
3. Clinical heterogeneity. VAP pathogenesis is heterogeneous. A gut-targeted metabolic intervention will unlikely benefit patients whose pneumonia is driven by massive, acute aspiration of highly virulent nosocomial pathogens independently of underlying host CIIP. Therefore, the clinical translation of this hypothesis demands precision medicine approaches—specifically, targeting gut-lung subphenotypes characterized by prominent early enteral feeding intolerance, barrier impairment, and subsequent immunosuppression (47).

## 4 Hypothesis testing

### 4.1 Molecular, laboratory, and advanced technological approaches

Validating the systemic nature of this hypothesis demands multi-omic and advanced laboratory techniques to capture the bottom-up physiological changes:

1. Microbial and metabolic profiling. Shotgun metagenomic sequencing of longitudinal fecal samples should be employed to assess structural shifts in the microbiome, moving beyond simple diversity indices to quantify the specific enrichment of SCFA-producing taxa (e.g., *Firmicutes*). Crucially, targeted metabolomics (e.g., gas chromatography–mass spectrometry, GC–MS) must be utilized to quantify circulating and fecal SCFA levels, directly testing the proposed metabolic signaling link to the lung.
2. Barrier integrity biomarkers. The severity of leaky gut and endotoxin translocation should be quantified using high-sensitivity laboratory assays for circulating structural markers, including intestinal fatty acid-binding protein (I-FABP), regenerating islet-derived protein 3-alpha (REG3α), lipopolysaccharide-binding protein (LBP), and sCD14-ST (presepsin) (48, 49).
3. Immune trajectory mapping. The transition from systemic hyper-inflammation to CIIP should be tracked using advanced flow cytometry to monitor monocytic HLA-DR (mHLA-DR) expression (19, 50, 51). Where feasible, single-cell transcriptomics could be applied to peripheral blood mononuclear cells (PBMCs) to decode the systemic immune reprogramming induced by the gut-targeted intervention.

### 4.2 Proposed ICU-feasible administration protocol and dose rationale

For mechanically ventilated patients, SLBZS should preferably be delivered as a standardized, Good Manufacturing Practice (GMP) grade granule or water-soluble extract, rather than as a crude decoction. Compared with traditional decoctions, standardized granules are easier to quantify by crude-drug equivalent, administer through a nasogastric or nasojejunal tube, and they may reduce the risk of tube obstruction and batch-to-batch variability. The total daily dose should be explicitly expressed as the equivalent mass of crude herbs rather than only as the mass of the finished granules.

Because no ICU-specific dose has been established, early feasible studies should use either an approved adult-label-equivalent dose or a conservative human-equivalent dose (HED) derived from preclinical data. If a rat dose is used as the starting reference, the HED should be calculated using body-surface-area normalization:

HED (mg/kg) = animal dose (mg/kg) × animal Km / human Km.

For rats and adult humans, commonly used Km factors are 6 and 37, respectively (52). The calculated HED should then be converted to a total daily crude-drug equivalent for a 60–70 kg adult and further reduced if safety margins are required for a first ICU feasibility study. The initial dose should be based on the approved adult clinical dose of the standardized SLBZS product used in the trial and should be reported as grams/day of crude-drug equivalent. If the specific commercial or hospital preparation differs in extraction ratio, the granule dose should be converted and reported according to the manufacturer’s certificate of analysis.

A preliminary protocol could initiate SLBZS within 24–48 h after intubation once enteral access has been confirmed and the patient is considered suitable for enteral administration. The target population should be patients expected to require invasive mechanical ventilation for more than 48–72 h, because VAP is conventionally defined as pneumonia occurring more than 48 h after endotracheal intubation or initiation of mechanical ventilation (2). In addition, epidemiological studies and reviews indicate that the risk of VAP is greatest during the early course of mechanical ventilation, particularly within the first 5 days, with a reported daily risk of approximately 3% during this period and a mean interval from intubation to VAP development of about 3.3 days (53). Treatment could continue for 5–7 days, until extubation, or until ICU discharge. In patients receiving antibiotics, SLBZS should be considered an adjunct to, not a substitute for, antimicrobial therapy or standard VAP prevention bundles.

SLBZS administration should be integrated with enteral nutrition protocols. In patients receiving continuous enteral feeding, SLBZS could be administered during scheduled medication windows, with tube flushing before and after administration. Nasojejunal delivery may be considered in patients with delayed gastric emptying or high aspiration risk. SLBZS should be withheld or discontinued in patients with suspected bowel ischemia, intestinal obstruction, severe ileus, uncontrolled gastrointestinal bleeding, abdominal compartment syndrome, persistent vomiting, severe or worsening feeding intolerance, rapidly escalating vasopressor requirements suggesting gut hypoperfusion, or suspected allergy to any component of the formula. Caution is also required in patients with severe hepatic or renal dysfunction, because the metabolism and safety profile of multi-component herbal preparations may differ from those in stable outpatient populations. Potential interactions with antibiotics, proton pump inhibitors, sedatives, vasopressors, anticoagulants, and enteral nutrition should be prospectively recorded in feasibility studies.

### 4.3 Clinical evaluation approach and control of confounding exposures

The clinical testing of this hypothesis should ideally encompass two phases. First, an observational phenotyping phase should be conducted in ventilated patients to define the gut-driven VAP phenotype. Baseline characterization should include not only gut-barrier and metabolic biomarkers, such as I-FABP, REG3α, LBP, sCD14-ST, fecal and circulating SCFAs, but also clinical gastrointestinal variables. These should include acute gastrointestinal injury (AGI) grade, feeding intolerance, gastric residual volume when used in the local ICU, vomiting, diarrhea, abdominal distension, bowel sounds, constipation or ileus, intra-abdominal pressure when clinically indicated, and the ratio of enteral to parenteral nutrition. Nutritional exposure should be recorded as daily caloric and protein delivery, enteral nutrition dose, parenteral nutrition dose, fiber exposure, and interruptions of feeding. To avoid misclassifying non-enterogenic VAP as gut-driven VAP, the phenotyping model should incorporate airway-related and infection-related variables, including aspiration risk, reintubation, tracheostomy, oral care bundle adherence, subglottic secretion drainage when applicable, baseline pneumonia status, antibiotic exposure, and respiratory microbiology. A gut-driven phenotype should therefore be defined by the co-occurrence of gastrointestinal dysfunction, barrier injury, dysbiosis or SCFA depletion, and immune-trajectory abnormalities, rather than by VAP occurrence alone.

Second, a prospective biomarker-stratified feasibility and interventional study should evaluate enteral SLBZS as an adjunct to standard ICU care. Major confounding medications and exposures should be prospectively recorded and controlled, including proton pump inhibitors, H2-receptor antagonists, broad-spectrum antibiotics, antifungal drugs, vasopressors, sedatives, opioids, corticosteroids, insulin, prokinetic agents, laxatives, probiotics, prebiotics, and enteral nutrition formulas. These variables should be handled through prespecified stratification, covariate adjustment, sensitivity analyses, and subgroup analyses. Patients with uncontrolled shock, suspected intestinal ischemia, severe ileus, or inability to receive enteral therapy should be excluded from interventional testing until safety is established.

### 4.4 Safety monitoring and pharmacovigilance

Gastrointestinal adverse events should include new or aggravated diarrhea, constipation, abdominal distension, vomiting, gastric retention, feeding intolerance, suspected aspiration, tube obstruction, and worsening ileus (27). Laboratory safety monitoring should include liver function tests, renal function, electrolytes, glucose, coagulation parameters when clinically indicated and inflammatory markers. Allergic reactions and unexpected hemodynamic deterioration should be recorded as potential adverse events.

Particular attention should be paid to interactions with antibiotics and other ICU medications (54). The bioavailability of orally or enterally administered drugs may be altered in critically ill patients (55). SLBZS may theoretically alter antibiotic pharmacokinetics by modifying gut microbiota-mediated metabolism, adsorbing drugs within the enteral lumen, or altering intestinal permeability. Therefore, early studies should record antibiotic class, route, dose, timing relative to SLBZS administration, therapeutic drug monitoring results when available, and clinical antibiotic response. For antibiotics with established therapeutic drug monitoring, such as vancomycin or aminoglycosides, serum concentrations should be compared between SLBZS-exposed and unexposed patients when feasible (56). Until these interactions are clarified, SLBZS should be separated from critical enteral medications by a predefined interval and administered with standardized tube flushing (57).

### 4.5 Falsifiable predictions

If our hypothesis is valid, we anticipate specific, directional shifts in the interventional cohort (Table 1). The hypothesis yields the following falsifiable predictions:

1. Barrier restoration. Evidenced by a rapid, statistically significant decrease in circulating I-FABP, REG3α, and sCD14-ST, alongside the normalization of LBP.
2. Ecological and metabolic recovery. Characterized by the metagenomic suppression of opportunistic pathogens (e.g., *Escherichia coli*), coupled with a measurable increase in systemic SCFA concentrations via metabolomics.
3. Immune reconstitution. Manifested as the accelerated recovery of mHLA-DR expression, preventing the onset of deep CIIP.
4. Clinical translation. A proportional reduction in VAP incidence that directly correlates with the degree of molecular and ecological recovery.

**Table 1:** Falsifiable predictions and alternative interpretations of the proposed SLBZS-mediated gut-lung axis hypothesis.

Pathway node	Readout(s)	Prediction if hypothesis holds	Negative prediction	Alternative interpretation
Gut-barrier injury	I-FABP; REG3α; zonulin; LBP; sCD14-ST	Barrier/translocation markers ↓	No biomarker improvement	Gut barrier not restored
Gastrointestinal dysfunction	AGI grade; feeding intolerance; gastric retention; EN/PN ratio	AGI grade ↓; feeding tolerance ↑	Persistent AGI III–IV or worsening feeding intolerance	Limited bedside feasibility
Dysbiosis dominance	α-diversity; Proteobacteria abundance	Diversity ↑; pathogen dominance ↓	No directional microbial shift	Antibiotics, PPIs, or critical illness override remodeling
Metabolic signaling	Fecal/circulating SCFAs	SCFAs increase or recover	SCFAs remain low, or increase without VAP reduction	Other independent pathogenic pathways exist
CIIP	Monocytic HLA-DR	Faster HLA-DR recovery	Immune trajectory unchanged or worsens	CIIP not reversed
Clinical outcome	VAP incidence; VFDs; antibiotic days	VAP risk ↓; VFDs ↑; antibiotic days ↓	No significant VAP-risk reduction	Non-enterogenic mechanisms dominate

↑ indicates increase or improvement; ↓ indicates decrease or reduction.

AGI, acute gastrointestinal injury; CIIP, critical illness-induced immunoparalysis; EN/PN, enteral nutrition/parenteral nutrition; HLA-DR, human leukocyte antigen-DR; I-FABP, intestinal fatty acid-binding protein; LBP, lipopolysaccharide-binding protein; PPIs, proton pump inhibitors; REG3α, regenerating islet-derived protein 3 alpha; SCFAs, short-chain fatty acids; sCD14-ST, soluble CD14 subtype/presepsin; SLBZS, Shenling Baizhu San; VAP, ventilator-associated pneumonia; VFDs, ventilator-free days.

Failure to observe these coordinated biological shifts in the mechanistic biomarkers, even if modest clinical improvements are noted, would challenge the proposed gut-lung causal pathway, suggesting alternative mechanisms of action.

## 5 Discussion

If the proposed hypothesis is validated, SLBZS could have potential health-economic value as a low-cost enteral adjunct, particularly in resource-limited settings. VAP is associated with prolonged mechanical ventilation, increased antibiotic exposure, longer ICU and hospital stays, and substantial additional healthcare costs. A host-directed intervention that safely reduces VAP susceptibility could therefore decrease antibiotic days, reduce ventilator duration, shorten ICU length of stay, and lower the burden of antimicrobial resistance and critical-care resource use.

The proposed mechanism also illustrates how a classical traditional Chinese medicine (TCM) formula can be translated into a modern critical-care hypothesis without reducing it to a single active compound. In TCM theory, the spleen governs transformation and transportation, and spleen qi deficiency with dampness may manifest as impaired digestion, fluid retention, phlegm accumulation, fatigue, and recurrent susceptibility to illness. In modern biomedical terms, these features can be cautiously mapped onto gastrointestinal dysfunction, impaired nutrient handling, dysmotility, barrier disruption, dysbiosis, and weakened host defense. SLBZS contains a core qi-tonifying and dampness-resolving structure, supported by herbs that regulate gastrointestinal function and facilitate phlegm clearance. Thus, its theoretical rationale aligns with a multi-target intervention aimed at restoring gut function and systemic resilience rather than directly killing respiratory pathogens. This framework helps explain why SLBZS is proposed as a preventive adjunct for a gut-driven VAP-susceptible phenotype, not as a stand-alone therapy for established pneumonia.

Beyond this conceptual shift, the proposed gut-lung mechanism yields specific predictions amenable to further clinical observation and experimentation. First, if enteral SLBZS successfully mitigates CIIP, prospective studies should demonstrate that gut-barrier restoration correlates with a generalized reduction in total nosocomial infection burdens, extending beyond VAP to include other ICU-acquired secondary infections (e.g., bloodstream infections). Second, this hypothesis implies that VAP susceptibility is heavily modulated by baseline gastrointestinal status, predicting that routine monitoring of gut-related biomarkers (such as serum zonulin or circulating SCFAs) could prospectively identify a gut-driven VAP sub-phenotype, thereby establishing a precision-medicine framework for targeted early interventions. Finally, confirming that dysbiosis directly fuels pulmonary vulnerability suggests that current standard ICU bundles require re-evaluation; future randomized controlled trials can test whether integrating microbiome-targeted botanical adjuncts, like SLBZS, into standard enteral nutrition yields superior long-term immunometabolic recovery compared to standard care alone.

In summary, we hypothesize that enteral SLBZS mitigates VAP risk via the gut-lung axis by reinforcing intestinal barrier integrity, reversing dysbiosis, and optimizing immunometabolic signaling to rescue patients from CIIP. Validating this host-directed strategy could provide health-economic value and expand VAP management from a predominantly pathogen-and airway-centered paradigm to a host-directed model focused on intestinal barrier integrity and microbial ecology.

## Data Availability

The original contributions presented in the study are included in the article/supplementary material, further inquiries can be directed to the corresponding author.
